# Chromosome-level genome assembly provides insights into the genetic diversity, evolution, and flower development of *Prunus conradinae*

**DOI:** 10.1186/s43897-024-00101-7

**Published:** 2024-06-19

**Authors:** Songtao Jiu, Muhammad Aamir Manzoor, Baozheng Chen, Yan Xu, Muhammad Abdullah, Xinyu Zhang, Zhengxin Lv, Jijun Zhu, Jun Cao, Xunju Liu, Jiyuan Wang, Ruie Liu, Shiping Wang, Yang Dong, Caixi Zhang

**Affiliations:** 1 Department of Plant Science, School of Agriculture and Biology, Shanghai Jiao Tong University, Shanghai, China; 2 Province Key Laboratory, Biological Big Data College, Yunnan Agricultural University, Kunming, China; 3 Shanghai Botanical Garden, Shanghai, People’s Republic of China

**Keywords:** *De *novo assembly, *Prunus conradinae*, Comparative genomic analysis, MADS-box genes

## Abstract

*Prunus conradinae*, a valuable flowering cherry belonging to the Rosaceae family subgenus *Cerasus* and endemic to China, has high economic and ornamental value. However, a high-quality *P. conradinae* genome is unavailable, which hinders our understanding of its genetic relationships and phylogenesis, and ultimately, the possibility of mining of key genes for important traits. Herein, we have successfully assembled a chromosome-scale *P. conradinae* genome, identifying 31,134 protein-coding genes, with 98.22% of them functionally annotated. Furthermore, we determined that repetitive sequences constitute 46.23% of the genome. Structural variation detection revealed some syntenic regions, inversions, translocations, and duplications, highlighting the genetic diversity and complexity of *Cerasus*. Phylogenetic analysis demonstrated that *P. conradinae* is most closely related to *P. campanulata*, from which it diverged ~ 19.1 million years ago (Mya). *P. avium* diverged earlier than *P. cerasus* and *P. conradinae*. Similar to the other *Prunus* species, *P. conradinae* underwent a common whole-genome duplication event at ~ 138.60 Mya. Furthermore, 79 *MADS*-box members were identified in *P. conradinae*, accompanied by the expansion of the *SHORT VEGETATIVE PHASE* subfamily. Our findings shed light on the complex genetic relationships, and genome evolution of *P. conradinae* and will facilitate research on the molecular breeding and functions of key genes related to important horticultural and economic characteristics of subgenus *Cerasus*.

**Supplementary Information:**

The online version contains supplementary material available at 10.1186/s43897-024-00101-7.

## Core

We successfully assembled a chromosome-scale genome of *P. conradinae*, detailing its genome structure, synteny information, evolutionary history, and WGD events. Structural variation detection revealed syntenic regions, inversions, translocations, and duplications, highlighting the genetic diversity and complexity of *Cerasus*. The expansion of the *SVP* subfamily, crucial for bud endodormancy and flowering time, suggests better adaptation to environmental changes. Our findings shed light on the complex genetic relationships and genome evolution of *P. conradinae*, supporting further research on the molecular breeding and key gene functions of subgenus *Cerasus*.

## Gene & Accession Numbers

The raw data of *Prunus conradinae* genome are available at figshare (10.6084/m9.figshare.25435240.v2).

## Introduction

The Rosaceae family comprises over 100 genera and approximately 3,000 species, including numerous fruit crops (e.g., apple, apricot, cherry, peach, pear, and strawberry), nuts (e.g., almonds), and ornamental plants (e.g., roses and flowering cherries) (Dirlewanger et al. 2002). The members of this family play crucial roles in providing nutritionally valuable foods and contributing significantly to the production of highly sought-after aesthetic and industrial products (Potter et al. 2007). In particular, *Cerasus*, a subgenus of *Prunus*, is a well-known horticultural plant resource of edible fruits and flowering trees (Wu et al. 2019). Indeed, China has the most abundant wild cherry germplasm resources globally, paving the way for the production of diverse hybrid varieties (Ma et al. 2009). However, frequent natural hybridization and selection processes often give rise to taxonomic controversies regarding the exact name, origin, and definition of various cherry germplasm resources, particularly of those in the wild (Jiu et al. 2023). Insufficient biological evidence and systematic classification can easily lead to confusion regarding the taxonomic groups of *Prunus* subg. *Cerasus*.

**Fig. 1:**
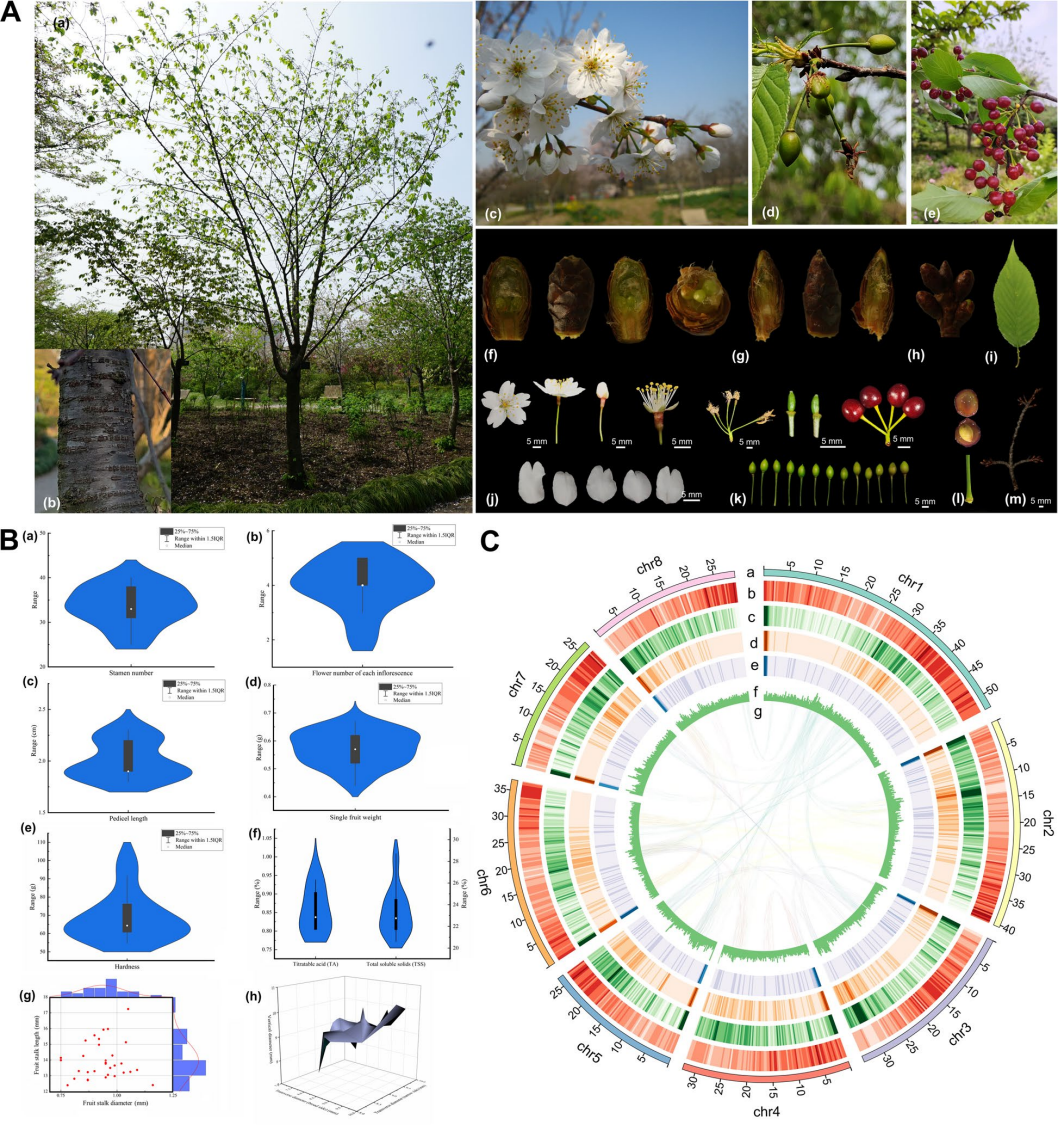
De novo genome assembly of *Prunus conradinae*. (A) Phenotypic characterization of *P. conradinae*. Various phenotypes were collected from February to May 2019, encompassing (a) individual panoramas, (b) barks, (c) blooming flowers, (d) green fruits, (e) ripe fruits, (f) flower buds, (g) leaf buds, (h) rosette buds, (i) leaves, (j) floral organs, (k) fruits of various stages, (l) fruit stalks, and (m) branches. (B) Flower and fruit parameters of *P. conradinae*, including (a) stamen number, (b) flower number of each inflorescence, (c) pedicel length, (d) single fruit weight, (e) hardness, (f) titratable acid and total soluble solids, (g) length and diameter of fruit stalks, and (h) vertical and transverse diameters of fruits. (C) Summary of the de novo genome assembly and sequencing analysis of *P. conradinae.* Moving from the outside to inside, the tracks indicate (a) chromosome size (Mb), (b) gene and (c) repeat density (300 kb sliding window), (d) *Gypsy* density (300 kb sliding window), (e) *Copia* density (300 kb sliding window), (f) GC content (300 kb sliding window), (g) synteny blocks among *P. conradinae* chromosomes

*Prunus conradinae* (Koehne) Yü et Li belonging to the *Cerasus* subgenus, is a wild flowering cherry plant with a high climatic adaptability and is widely distributed throughout China, where it is endemic to many provinces, including Fujian, Guangxi, Henan, Guangxi, Yunnan, Hubei, Guizhou, Sichuan, Hunan, Shanxi, and Zhejiang (Fu et al. 2016; Wu et al. 2019). The species is typically found in forests and valleys, flourishing at altitudes ranging from 500 to 2100 m (Wu and Raven 2003).  Highly esteemed for its ornamental value, the tree is adorned with resplendent white or pink flowers that are predominantly produced from March to April and have a striking appearance. Umbels typically bear 3–5 flowers with ovate or obovate petals and approximately 25–43 stamens that are nearly as long as the petals, pedicels extending 1.8–2.3 cm in length, and red ovoid fruits (Fig. 1A-B, Table S1; Wu and Raven 2003). In certain warm climates, flowering can begin as early as January, and the tree can grow up to 10 m, bearing leaves with light-green abaxial and dark-green adaxial surfaces (Yu and Li 1986; Wang 2014). In recent years, horticulturists have developed *P. conradinae* cultivars with unique flower shapes, petal colors, and strong aromas; for example “Longyun” and “Chujin” (Lura and Whittemore 2021; Dong et al. 2020; Jiang et al. 2022). As an important species for cherry breeding, *P*. *conradinae* has potential to be used for cross-breeding to select high ornamental-value flowering cherries and excellent cherry rootstock varieties suitable for the climate and soil conditions in China, because of its compatibility with other species in subgenus *Cerasus* (Dong et al. 2020). Nevertheless, surprisingly, few investigations have been conducted on *P. conradinae*, particularly phylogenetic analyses, resulting in a relatively unknown genetic background and, consequently, the neglect of molecular marker use and mining for key genes that regulate important traits.

Owing to their early flowering and superior ornamental value, flowering cherries have gradually become popular decorative plants worldwide and subjects of increased research interest. However, the genetic background of this important species and particularly the genetic factors regulating its flower development remain relatively unknown. MADS-box proteins are an important regulatory factors that control flowering transition and floral organ development in flowering plants (Smaczniak et al. 2012). These factors are divided into two major lineages (type I and II) based on their distinct protein domains (De Bodt et al. 2003; Henschel et al. 2002; Kofuji et al. 2003). Type I proteins, which are encoded by M-type genes, are subdivided into Mα, Mβ, and Mγ categories, while Type II proteins contain the MIKC domain and are further divided into MIKC^C^- and MIKC*-types (Henschel et al. 2002; Jiu et al. 2023). In *Arabidopsis thaliana* (Atha), these factors have a decisive influence on developmental processes, including, leaf morphogenesis, growth, and seed and flower development (Becker and Theißen 2003). *Dormancy-associated MADS-box* (*DAM*) genes and other orthologs of *SHORT VEGETATIVE PHASE* (*SVP*) gene, belonging to the *SVP*/*AGAMOUS-LIKE 24* (*AGL24*) subfamily, are involved in regulating flowering time and bud dormancy (Gao et al. 2021; Wang et al. 2020a; Bielenberg et al. 2008). Therefore, elucidating the mechanisms underlying the control of flowering time involving MADS-box family genes might help address flowering anomalies presumably caused by climate change.

The availability of genome assemblies for *Prunus* species has long been limited by their high degree of heterozygosity, which has impeded research on topics, such as desirable traits and genomic organization. In recent years, the rapid development of next-generation sequencing (NGS) technologies has enabled the assembly of high-quality genomes of some *Prunus* species with extremely heterozygous genetic backgrounds. The* P. mume* (Pmum) genome was sequenced and published in 2012, becoming the first *Prunus* fruit genome available (Zhang et al. 2012). Subsequently, several genomes from subgenus *Cerasus* have been sequenced, including *P. yedoensis* (Pyed; Baek et al. 2018), a *Cerasus* × *yedoensis* hybrid (“Somei-Yoshino”) (Shirasawa et al. 2019), *P. avium* (Pavi; Wang et al. 2020b), *C. serrulata* (Yi et al. 2020), *P. fruticosa* (Pfru; Wöhner et al. 2021), *P. pusilliflora* (Ppus; Jiu et al. 2023), and *P. campanulata* (Pcam; Nie et al. 2023). These genomic resources have greatly enhanced our understanding of the origin, evolution, and genomic selection of *Cerasus*. Considering that high-quality genome assemblies have contributed to clarifying the phylogenetic relationships of various *Cerasus* species and resolving their taxonomic controversies, there is still need for higher-quality chromosome-scale genomes for other species in this subgenus. However, to date, whole-genome sequencing and chromosome-level assembly of the *P. conradinae* genome have not been reported. Therefore, in this study, we assembled a high-quality chromosome-level *P. conradinae* genome and compared it with the publicly available genomes of *Cerasus*. In addition, we investigated the *MADS*-box family of *P. conradinae* as well as the genetic diversity, structural variation, and phylogenetic hierarchy of the species in relation to other *Prunus* species. The newly assembled *P. conradinae* genome provides a resource that will facilitate research on the molecular breeding and the functions of key genes related to important horticultural and economic characteristics of subgenus *Cerasus*.

## Results

### Genome sequencing, assembly, and annotation of *P. conradinae*

We obtained 74.36 Gb of Illumina short-read data and 44.97 Gb of Oxford Nanopore Technology (ONT) long-read data (Table S2). The haploid genome size (266.84 Mb) of *P. conradinae* was estimated using flow cytometry (Figure S1 and Table S3). After obtaining the draft genome, we conducted chromosome-level assembly using 90.91 Gb of high-throughput chromosome conformation capture (Hi-C) reads. After correcting the chromosome order and direction, the chromosome-level genome assembly contained 26 scaffolds, covering 289.62 Mb, with a contig N50 of 4.47 Mb and scaffold N50 of 34.17 Mb (Fig. 1C, Table S4). Benchmarking Universal Single-Copy Ortholog (BUSCO) analysis indicated 97.1% completeness, with only 2.3% missing BUSCOs (Table S5). In total, 279.87 Mb (~ 96.63%) of the genome was anchored to eight pseudochromosomes (Table S6). Furthermore, the Hi-C heatmap did not reveal any notable assembly errors among the eight pseudochromosomes, which were well-connected along the diagonal line (Figure S2). We identified 129.38 Mb repetitive sequences (~ 46.23% of the genome), including tandem repeats and transposable elements (TEs) (Table S7). The most abundant TEs were Long terminal repeat retrotransposons (LTR-RTs), accounting for 25.88% (Table S7). Most of the LTR-RTs were LTR/*Gypsy* and LTR/*Copia* elements, accounting for 14.98% and 10.05% of the total, respectively, which greatly expanded the genome (Table S7). Among the TEs, DNA transposons accounted for 10.29% of the haploid genome (Table S7). Collectively, these results strongly indicate that TE insertions have been mainly responsible for genomic expansion in *P. conradinae*.

We identified 31,134 protein-coding genes in the *P. conradinae* genome, which were supported by *ab initio*, homologous, and de novo predictions. A comparison between the *P. conradinae* (Pcon) genome (97.1%) and the annotated gene set (94.2%) revealed that their BUSCO completeness was similar, indicating that most genes in the *P. conradinae* genome were successfully annotated (Table S8). Specifically, 30,580 (98.22%) genes were functionally annotated via searches of non-redundant (NR; 30,570 genes), Swiss-Prot (21,205), eggNOG (26,018), Gene Ontology (GO; 9,742), Clusters of Orthologous Groups of proteins (COG; 26,018), the Arabidopsis Information Resource (TAIR; 24,116), Kyoto Encyclopedia of Genes and Genomes (KEGG; 12,084), and Pfam (21,017) databases (Table S9). In addition, LTR analysis showed that long terminal repeat assembly index (LAI) of the *P. conradinae* genome (18.26) was only slightly lower than those of the well-assembled Pavi (19.68) and *P. persica* (Pper; 18.79) genomes but higher than those of the Ppus (17.35), *P. armeniaca* (Parm; 16.29), Pyed (6.87), and *P. domestica* (2.27) genomes, underscoring the superior assembly quality of the *P. conradinae* genome (Table S10).

### Syntenic analysis and structural variation detection between *P. conradinae* and other *Prunus* species

**Fig. 2:**
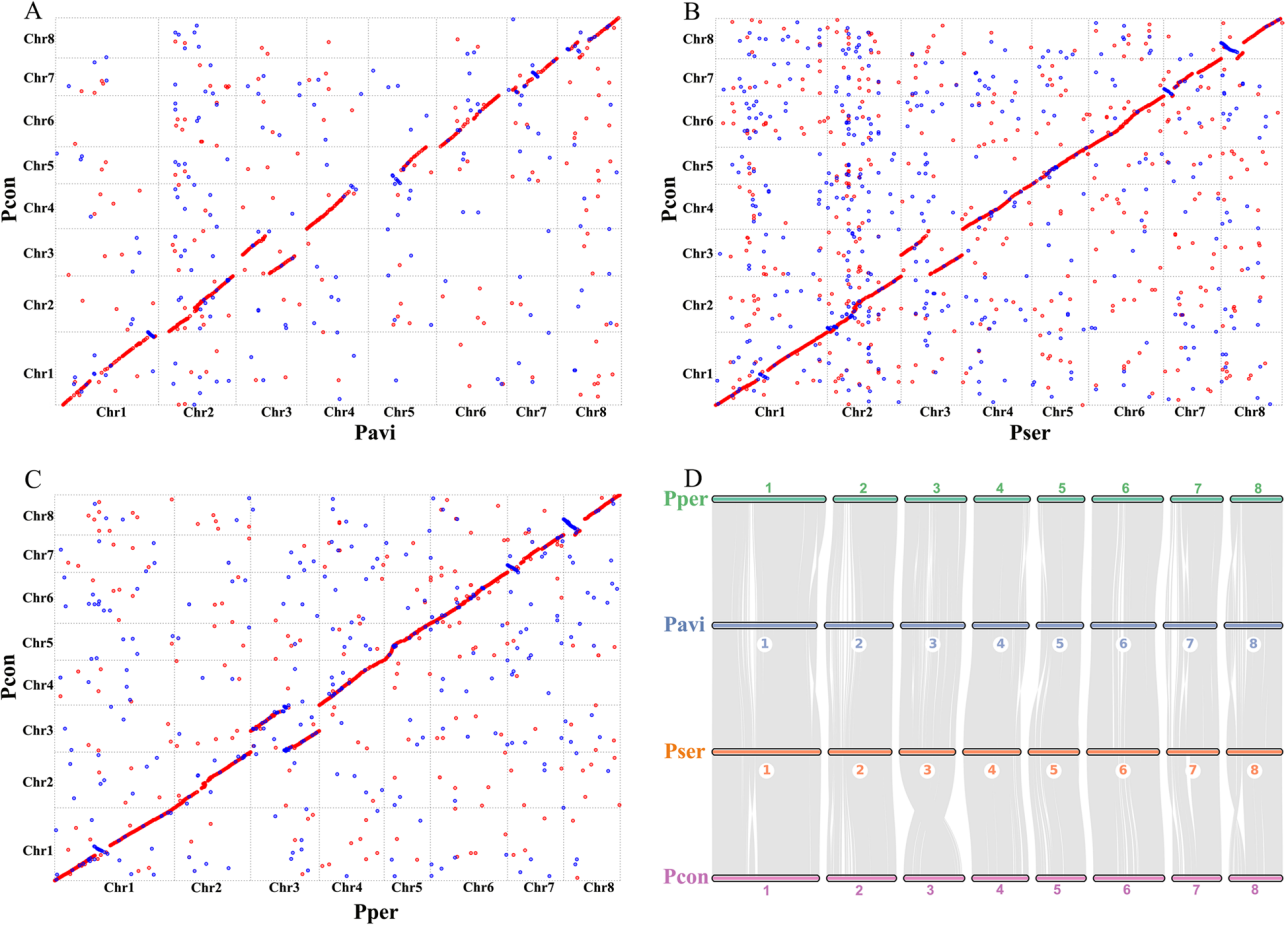
Synteny analysis of *Prunus conradinae*, *P. avium*, *P. serrulata* and *P. persica*. Synteny maps comparing the *P. conradinae* genome with the (**A**) *P. avium*, (**B**) *P. serrulata*, and (**C**) *P. persica* genomes. Red and blue denote similar sequences in the same and opposite orientations, respectively. (**D**) Syntenic blocks among *P. conradinae*, *P. avium*, *P. serrulata*, and *P. persica*. Numbers represent the chromosome order from the original genomic sequence. Each line represents one block. Pcon: *P. conradinae*; Pavi: *P. avium*; Pser: *P. serrulata*; Pper: *P. persica*; Chr 1–8: chromosomes 1–8

We conducted a detailed syntenic analysis to elucidate the collinearity between Pcon and various *Prunus* species, generating synteny maps after comparing the Pcon genome with those of Pavi, Pper, and *P. serrulata* (Pser) (Fig. 2A-C). The synteny maps showed that Pcon has strong collinear relationships with Pavi, Pser, and Pper, with 1,086, 2,731, and 4,622 syntenic blocks, respectively (Tables S11–13). The statistical results displayed 261 and 825 syntenic blocks on different and same chromosomes in Pcon *vs*. Pavi synteny map, respectively (Table S14). The gene syntenic blocks from the comparison of the four *Prunus* genomes were distributed across eight chromosomes, indicating robust cross-species synteny (Fig. 2D). We observed all syntenic blocks located on the same chromosome (Tables S15–17), indicating that *P. conradinae* was closely related to *P. avium* and *P. serrulata*.

**Fig. 3:**
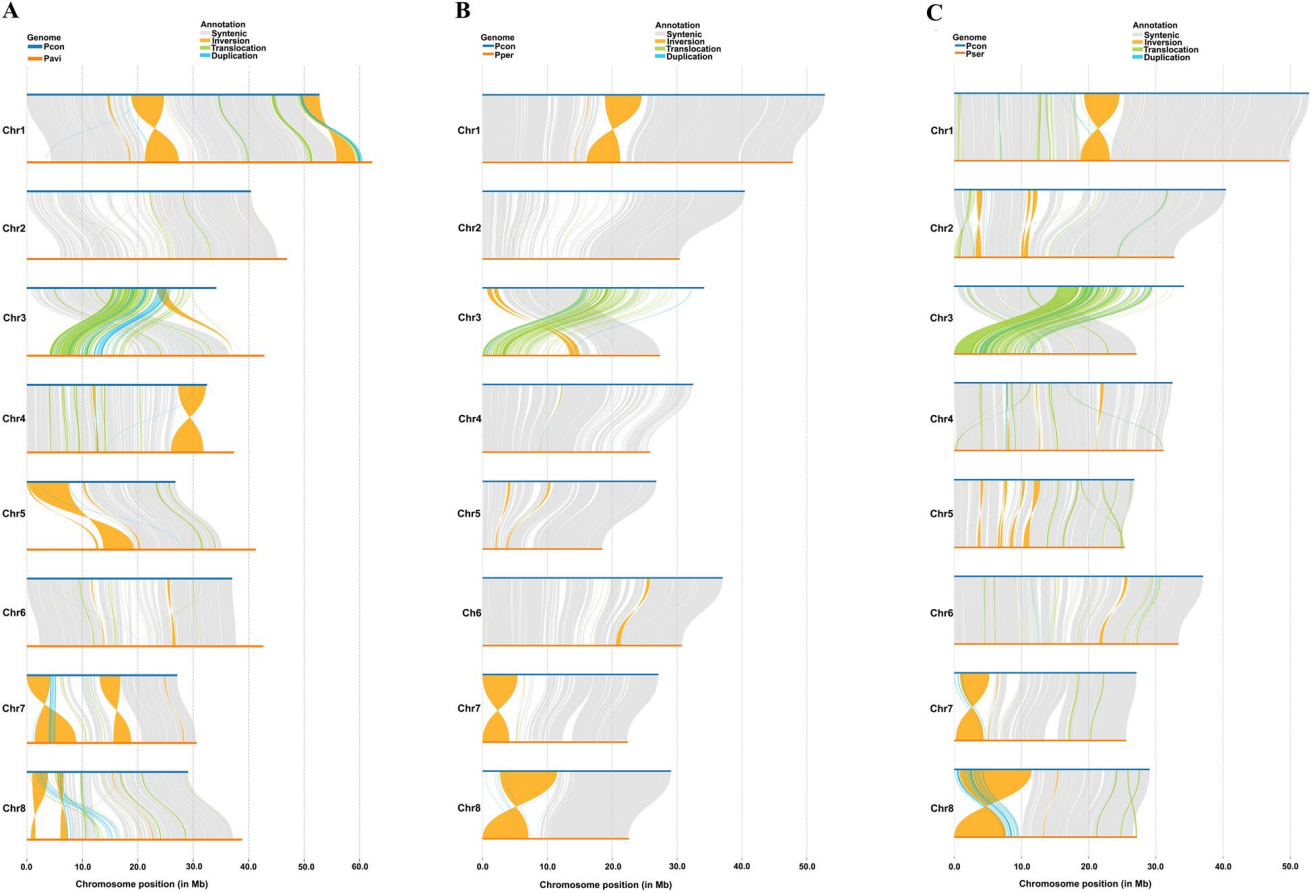
Structural variation detection between the *Prunus conradinae* genome and *P. avium* (**A**), *P. persica* (**B**), and *P. serrulata* genomes. (**C**). Pcon: *P. conradinae*; Pavi: *P. avium*; Pser: *P. serrulata*; Pper: *P. persica*; Chr 1–8: chromosomes 1–8

To compare structural variations between Pcon genome and those of multiple *Prunus* species, we identified syntenic regions, inversions, translocations, duplications, and unaligned genomic segments using MUMmer v.3.23 and Synteny and Rearrangement Identifier (SyRI) (Fig. 3; Goel et al. 2019). Our findings revealed significant syntenic regions between Pcon and each of the compared species (163.70 Mb for Pavi, 183.43 Mb for Pper, and 192.19 Mb for Pser), indicating their evolutionary conservation (Tables S18–20). Additionally, we identified genomic rearrangements with each comparison, including inversions (38.17 Mb for Pavi, 22.87 Mb for Pper, and 26.20 Mb for Pser), translocations (15.15 Mb for Pavi, 5.44 Mb for Pper, and 16.36 Mb for Pser), and duplications (3.10 Mb for Pavi, 1.04 Mb for Pper, and 2.20 Mb for Pser), suggesting the existence of mechanisms for species differentiation and adaptation (Tables S18–20). A notable aspect of our analysis was the considerable portions of the genome that remained unaligned in each comparison (66.70 Mb for Pavi, 68.04 Mb for Pper, and 49.01 Mb for Pser), highlighting the genetic diversity and complexity among these species (Tables S18–20). These findings contribute to our understanding of the genomic architecture and the evolutionary relationships within and between these species, underlining the importance of genomic rearrangements in species evolution and adaptation.

### Phylogenetic and whole genome duplication (WGD) event analysis

**Fig. 4:**
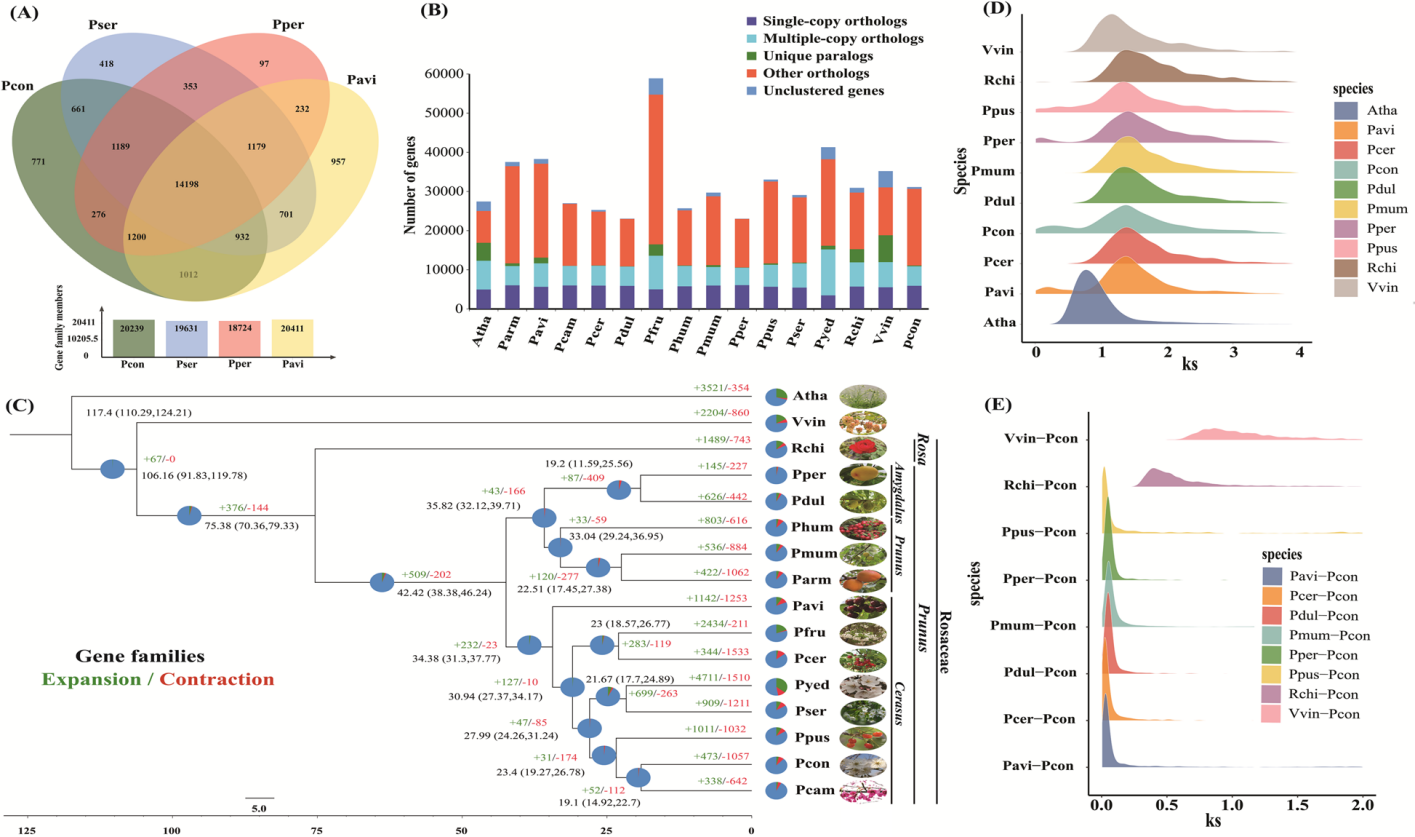
Comparative analysis of gene families between the genome of *Prunus conradinae* and those of other species. **A** Venn diagram showing shared and unique gene families among four *Prunus* genomes. **B** Gene number distribution of single-copy, multiple-copy, and other orthologs as well as unique paralogs, and unclustered genes in *A. thaliana* (Atha), *P. armeniaca* (Parm), *P. avium* (Pavi), *P. campanulata* (Pcam), *P. cerasus* (Pcer), *P. dulcis* (Pdul), *P. fruticosa* (Pfru), *P. huminis* (Phum), *P. mume* (Pmum), *P. persica* (Pper), *P. pusilliflora* (Ppus), *P. serrulata* (Pser), *P. yedoensis* (Pyed), *Rosa chinensis* (Rchi), *V. vinifera* (Vvin), and *P. conradinae* (Pcon). **C** Phylogenetic tree, divergence time, and profiles of contracted and expanded gene families in Pcon and 15 other plant species. **D** Synonymous substitution rates (*Ks*) for 10 plant species, including Atha, Vvin, Rchi, Ppus, Pper, Pmum, Pdul, Pcon, Pcer, and Pavi. **E** *Ks* distribution of orthologous gene pairs from Pcon compared with those of orthologous gene pairs from Vvin, Rchi, Ppus, Pper, Pmum, Pdul, Pcer, and Pavi

We identified 20,239 gene families in Pcon, more than the number in Pper and Pper and less than that in Pavi (Fig. 4A). The four *Prunus* species shared 14,198 gene families, while Pcon contained a higher number of unique gene families (771) than those in Pser (418) and Pper (97) (Fig. 4A). We then compared the number of unique paralogs, multiple- and single-copy orthologs, other orthologs, and unclustered genes between Pcon and the 15 selected species (Fig. 4B; Table S21). A total of 473 and 1,057 gene families expanded and contracted, respectively, in Pcon after speciation from Pcam (Fig. 4C). The numbers of expanded and contracted gene families was lower than those of other *Cerasus* species (Pser, Pyed, and Pavi). The expanded, contracted, and unique gene families were significantly enriched (*P* < 0.05) in 521, 81, and 204 GO terms, respectively (Supplementary Tables S22–24). Specifically, expanded genes were the significantly enriched in the sorbitol, mannitol, pentose, galactose, and glycerol transmembrane transport processes (Figure S3), the contracted genes were significantly enriched in proanthocyanidin biosynthetic and melatonin biosynthetic processes (Figure S4), whereas the unique genes were significantly enriched in maintenance of floral organ identity and pollen maturation processes (Figure S5).

To investigate genome evolution, we compared Pcon with 15 other plant species, using Atha and *V. vinifera* (Vvin) as outgroups. We used 1,079 single-copy genes from 16 plant species to construct a maximum-likelihood (ML) phylogenetic tree and found that Pcon was a sister species to Pcam and was closely related to the released Ppus (Fig. 4C). Furthermore, one branch comprising three *Cerasus* species (Pcon, Pcam, and Ppus) and another branch comprising two *Cerasus* species (Pyed and Pser) were clustered with two other *Cerasus* species (Pfru and Pcer) on a separate branch, followed by Pavi from *Cerasus* (Fig. 4C). Two subgenus *Amygdalus *species (Pper and Pdul) clustered with three subgenus *Prunus *species (Phum, Pmum, and Parm) and were closely related to all the subgenus *Cerasus* species (Pavi, Pfru, Pcer, Pyed, Pser Ppus, Pcon, and Pcam) (Fig. 4C). Based on the fossil calibration of know species in the TimeTree database (http://www.timetree.org/), we determined the time when Pcon and other plant species diverged. The divergence of Pcon and Pcam was estimated to have occurred at ~ 19.10 Mya (95% HPD of 14.92–22.70 Mya). The five *Cerasus* species (Pyed, Pser Ppus, Pcon, and Pcam) split from the two *Cerasus* species (Pfru and Pcer) approximately 30.94 Mya (95% HPD of 27.37–34.17 Mya).

Positively selected gene pairs for Pavi *vs.* Pcon, Pcon *vs.* Pper, and Pser *vs*. Pcon were numbered 181, 15, and 723, respectively (Tables S25–27). We identified 12, 48, and three positively selected genes encoding transcription factors (TFs) with matched Pfam domains in the Pavi *vs.* Pcon, Pser *vs*. Pcon, and Pcon *vs.* Pper, respectively (Tables S28-30). Functional analysis of common TFs (e.g., NAC, ERF, MYB, bHLH, bZIP, and WRKY) indicated that they are more likely to participate in *P. conradinae* growth and development, and its stress response process. We compared the distribution of synonymous substitution rates (*Ks*; Fig. 4D) to investigate WGD events in the Pcon genome. The *Ks* distribution of Pcon showed a clear peak at ~ 1.376, similar to that of other selected Rosaceae species, indicating that Pcon experienced a common WGD event in the Rosaceae family (Fig. 4D; Table S31). Referring to the WGD event of Vvin (117 Mya) (Jiao et al. 2012), we estimated that the WGD event of *P. conradinae* occurred at ~ 138.60 Mya (Table S31). We then used *Ks* distributions of orthologous genes to deduce the time of divergence of the Pcon genome from the angiosperm genomes (Fig. 4E). Pcon showed a single peak with Pcer, Pavi, Pdul, Pper, Pmum, and Rchi at *Ks* values of 0.0215, 0.0324, 0.0418, 0.0435, 0.0473, and 0.4218, respectively (Fig. 4E; Table S32). From these data, we inferred that the diversification of the five *Prunus* species occurred recently. In addition, *P. avium* diverged earlier than *P. cerasus* (Pcer) and Pcon did (Fig. 4C, E).

### Identification and phylogenetic analysis of the MADS-box gene family in *P. conradinae*

**Fig. 5:**
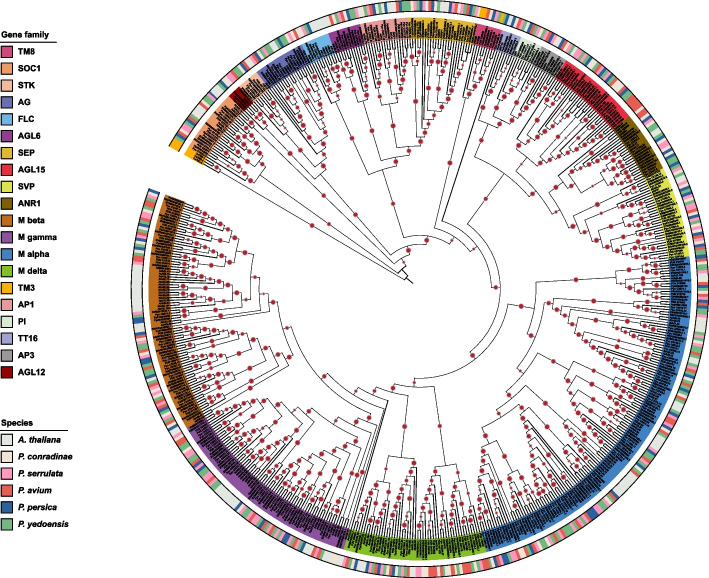
Phylogenetic analysis of MADS-box gene family members in *Prunus conradinae.* Grapevine TOMATO MADS-box 8 (TM8) (TC62855), poplar TM8 (XP_002321711.1), and *P. mume* PmMADS26 (Pm024524) were used for the phylogenetic analysis because the *Arabidopsis* genome lacks the TM8 subfamily. The *Coffea arabica* TOMATO MADS-box 3 (TM3), CaTM3-1 (KJ483226), CaTM3-2 (KJ483227), and CaTM3-3 (KJ483228) were also used to construct the phylogenetic tree

MADS-box family genes have been reported in multiple *Prunus* species (Xu et al. 2014; Wells et al. 2015, Jiu et al. 2023). However, a detailed characterization of this gene family in *P. conradinae* has not been previously reported. Herein, 79 MADS-box members were identified in the Pcon genome (Table S33). In addition, in accordance with the classification for Atha, we categorized the type I MADS-box genes in Pcon into four distinct groups: M-alpha with 16 genes, M-beta with 13 genes, M-delta with eight genes, and a smaller group, M-gamma, with five genes (Fig. 5). On the basis of the phylogenetic analysis results, the type II MADS-box genes in Pcon were divided into 15 notable subfamilies: SUPPRESSOR OF OVEREXPRESSION OF CONSTANS 1 (SOC1), SVP, TOMATO MADS-box 8 (TM8), AGL15, ARABIDOPSIS NITRATE REGULATED 1 (ANR1), AGL6, SEPALLATA (SEP), PISTILLATA (PI), APETALA3 (AP3), SEEDSTICK (STK), AGAMOUS (AG), APETALA1 (AP1), FLOWERING LOCUS C (FLC), AGL12, and TRANSPARENT TESTA 16 (TT16)/AGL32 (Fig. 5). Notably, 14 of these subfamilies were congruent with their counterparts in *Arabidopsis*, suggesting evolutionary conservation across these species. We used *TM8* (TC62855) in Vvin, *TM8* (XP_002321711.1) in poplar, a homologous gene (*PmMADS26*) of *TM8* in Pmum, and CaTM3-3 (KJ483228), CaTM3-2 (KJ483227), and CaTM3-1 (KJ483226) in *Coffea arabica* for the phylogenetic analysis because the *Arabidopsis* genome lacks the TM3 and TM8 subfamilies (Xu et al. 2014; Heijmans et al. 2012; Díaz-Riquelme et al. 2009). The Pcon *MADS-*box gene (PAV05G010960.1) was unambiguously grouped with these three TM8 genes (Fig. 5), indicating that the Pcon genome has only one TM8 member, similar to poplar, Pmum and grapevine. Similar to the *Arabidopsis*, Pyed, Pser, and Pper (Jiu et al. 2023), the *P. conradinae* genome appeared to lack members of TM3 subfamily, indicating that this subfamily might be unique to *C. arabica*. The SVP (7) and AGL15 (3) are the expanded type II MADS-box subfamilies in *P. conradinae* compared with those in *Arabidopsis*. Moreover, the collinearity analysis revealed 79 pairs of collinear MADS-box genes between *P. conradinae* and *P. serrulata* (Table S34). This comprehensive analysis not only enhances our understanding of the MADS-box gene family in *Prunus* species but, additionally, opens avenues for further research into the evolutionary and functional characterization of these genes, particularly in plant development and adaptation.

## Discussion

*P. conradinae* of subgenus *Cerasus*, is a commercially important flowering plant with high economic and ornamental value in China (Shang et al. 2022). However, the high-quality assembly of *Cerasus* genomes has been hampered owing to their high degree of heterozygosity, thus limiting our understanding of the heterosis, trait inheritance, and genomic evolution of the species in *Cerasus*. Therefore, there is an urgent need for comprehensive whole-genome sequencing data to facilitate the conservation and utilization of valuable genetic resources within this subgenus. Herein, the genome assembly for *P. conradinae* is reported for the first time. We found that the assembled Pcon genome (289.62 Mb) was smaller than those of Pyed (323.8 Mb) (Baek et al. 2018), Pavi (344.3 Mb) (Wang et al. 2020b) and Pfru (366.5 Mb) (Wöhner et al. 2021), but larger than those of Pser (265.4 Mb) (Yi et al. 2020) and Pcam (280.2 Mb) (Wöhner et al. 2021). Moreover, the *P. conradinae* genome had a lower repetition rate (~ 46.23%) than those of the Pyed (~ 47.20%) (Baek et al. 2018), Pavi (~ 59.40%) (Wang et al. 2020b), and Pfru (~ 51.75%) (Wöhner et al. 2021) genomes. The results suggest that the relatively low repetition rate might be the main reason *P. conradinae* has a smaller genome size than those of the other three species. In addition, scaffold N50 (34.17 Mb) and contig N50 (4.47 Mb) values of Pcon were higher than those of Pyed (Baek et al. 2018) and Pser (Yi et al. 2020) but comparable to those of Ppus (Jiu et al. 2023). This novel genome assembly offers valuable resources for cherry breeding and investigating the genetic diversity and evolution of the subgenus *Cerasus*.

Phylogenetic analysis unveiled a distinct clustering pattern among species belonging to the subgenus *Cerasus*, including Pcon, Pavi, Pfru, Pcer, Pyed, Pser, Ppus, and Pcam. These species exhibited the shortest divergence time and formed an independent branch, clearly demarcating them from species within the subgenus *Prunus*, such as Phum, Pmum, and Parm, as well as from the subgenus *Amygdalus* species, such as Pper and Pdul. Consistent with previous findings (Jiu et al. 2023; Baek et al. 2018; Yi et al. 2020), our findings indicated that Pavi diverged earlier than Ppus, Pser and Pyed did. Our investigation revealed that, similar to other species within the Rosaceae, *P. conradinae* underwent a common WGD event. As it is a tetraploid species (Wang et al. 2023), we propose that, in addition to this WGD event shared with other members of the Rosaceae family, *P. conradinae* has experienced a lineage-specific polyploidy event. However, predicting this event based solely on homologous genes remains challenging. We observed minor peaks in the regions where the *Ks* distribution closely approached zero across several species (Ppus, Pper, Pcon, and Pavi). These peaks might be attributed to fragmentation or repetition within the genomes of these species (Fig. 4D). Case in point, we observed possible structural variation on chromosome 3 between the Pcon and Pavi/Pser/Pper genomes (Fig. 2). One possible explanation involves a large translocation between the genomes of three *Prunus *species (Pavi/Pser/Pper) and that of Pcon, or perhaps there is a problem with the mounting of Pcon at the chromosome level. Further investigation is warranted to validate these inversions conclusively.

Previous reports have revealed that the occurrence of polyploid plants in nature is not random but primarily influenced by ecological and climatic factors (Hohmann and Koch 2017; Ren et al. 2018). *P. conradinae* is an important cherry germplasm resource with high climatic adaptability and wide distribution in China. In this study, the expanded gene families were observed to be significantly enriched in plant process terms related to the transmembrane transport of sugars and alcohols (GO:0015750, GO:0015752, GO:0015753, GO:0015795, GO:0015797, GO:0015757, GO:0015793, GO:0015791), jasmonic acid and ethylene-dependent systemic resistance (GO:0009861), and lateral root morphogenesis (GO:0010102), and formation (GO:0010311). The unique gene families were observed to be significantly enriched in maintenance of floral organ identity and pollen maturation processes. These findings underscore the pivotal roles of these genes in regulating plant growth, development, and adaptation to varying environmental conditions. Given the significance of *P. conradinae* as an early spring ornamental tree species, we focused on the investigation on the MADS-box gene family due to its involvement in floral organ development and dormancy release. We identified 79 *MADS*-*box* genes in *P. conradinae*, commonly known as flowering cherry, which is fewer than those in Pyed and Pser (Jiu et al. 2023). The expansion of the *SVP* subfamily in Pcon, associated with flowering time, indicates an evolutionary adaptation toward a more precise control of flowering time. Furthermore, *DAM* genes, often referred to as *SVP* or *SVP*-like (*SVL*), are known to play a role in inhibiting bud break in pears (Gao et al. 2021). Our findings indicate that seven Pcon *MADS*-box genes align closely with two *AtSVP* members (Fig. 5), highlighting their importance in regulating bud endodormancy.

In conclusion, we first assembled a high-quality haploid genome for *P. conradinae* using Illumina, ONT, and Hi-C technologies. This represents the initial step toward gaining a comprehensive understanding of the molecular foundations governing diverse desirable traits within economically significant *Cerasus* species, although chromosomal structural diversity and haplotype-resolved genomes warrant further research. Nonetheless, our findings lay the foundation for future research in the fields of comparative genomics, molecular biology, molecular breeding, genetics, and evolutionary aspects of the species in subgenus*Cerasus*.

## Materials and methods

### Plant materials and DNA extraction

 Fresh young leaves were harvested from a 15-year-old *P. conradinae* tree at Shanghai Botanical Garden, Shanghai, China (121° 27ʹ 4ʺ N, 31° 9ʹ 14ʺ E). Stamen number, flower number of each inflorescence, pedicel length, fruit weight, total soluble solids, length and diameter of fruit stalks, and vertical and transverse diameters of fruits were determined in this study. The hardness of *P. conradinae* fruits was evaluated using the TA.XT Plus Texture Analyzer (Stable Micro Systems, Surrey, UK) with the following parameters: P/50 flat probe, pre-test speed of 5 mm/s, post-test speed of 5 mm/s, a pause time between cycles of 5 s, a trigger force of 5 g, and test speed of 0.5 mm/s. Titratable acid of ripe fruits was measured using the method described by Kazemi et al. (2011), with the value expressed as the percentage of anhydrous malic acid. High-purity genomic DNA was extracted using the DNeasy Plant Kit (Tiangen Biotech Co. LTD, China). The purity and concentration of the extracted DNA were meticulously assessed using a Nanodrop 2000 spectrometer (Thermo Fisher Scientific, Waltham, MA, USA) and a Qubit® 3.0 fluorometer (Thermo Fisher Scientific Inc.). DNA integrity was evaluated by means of 0.8% agarose gel electrophoresis using the pulsed-field technique.

### Genomic DNA sequencing

A combination of long- and short-read sequencing data was used to assemble the *P. conradinae* genome. A paired-end library was constructed for Illumina short-read sequencing, using the GenElute Plant Genomic DNA Miniprep Kit (Sigma-Aldrich, Corp., St. Louis, MO, USA). This construct was then sequenced on an Illumina HiSeq X Ten platform (Illumina Inc., San Diego, CA, USA). A total of 2 µg DNA was used for the ONT library construction. After the sample was qualified, long DNA fragments was selected using the BluePippin system (Sage Science, Beverly, MA, USA). Further, the ends of DNA fragments were repaired and a ligation reaction was conducted using the NEBNext® Ultra™ II End Repair/dA-Tailing Module Kit. The ONT sequencing library was prepared using the ligation sequencing kit 1D (SQK-LSK109; Oxford Nanopore Technologies, Oxford, UK) raccording to the manufacturer’s instructions. Qubit® 3.0 fluorometer was used to quantify the size of library fragments. The ONT sequencing was then performed on an Oxford Nanopore PromethION 48 platform at Novogene Co., Ltd. (Beijing, China). A Hi-C sequencing library was constructed via chromatin extraction and digestion followed by DNA ligation, purification, and fragmentation (Belton et al. 2012), before sequencing on the Illumina HiSeq X Ten platform .

### Genome assembly and evaluation

Before de novo assembly, we evaluated the genome size of *P. conradinae* by means of flow cytometry (BD FACSCalibur), using tomato as the internal standard. Fastp v.0.20.2 (Chen et al. 2018) was used to perform quality control of the NGS data, including Hi-C reads, RNA-Seq data, and whole genome sequencing paired-end reads, with default parameters to produce clean reads. For the Nanopore data, passed reads were assembled into a de novo genome using NECAT v.0.0.1 (https://github.com/xiaochuanle/NECAT) with default parameters (Chen et al. 2021), and then polished three iterations using Racon with default parameters (Vaser et al. 2017). All clean Illumina paired-end reads were adapted to polish two iterations using Pilon v.1.21 with default parameters (Walker et al. 2014). Subsequently, redundant sequences were removed using purge_dup v.1.2.5 with default parameters and the final contig genome was produced. Hi-C data allow for the correction of assembly errors, complement reads, and optical maps to improve the scaffolding of contigs and provide chromosome-spanning contiguity for the assembly. Clean Hi-C data were utilized for chromosome-level genome assembly using HiC-Pro (Servant et al. 2015) and 3D-DNA v.180922 (Dudchenko et al. 2017) with default parameters. Based on the Hi-C heatmaps, the chromosome-level genome assembly was manually checked for misorientation using Juicer v.1.6.2 (Durand et al. 2016). Then, NGS data were mapped to the assembly using the Burrows-Wheeler Aligner with default settings, yielding an estimate of the coverage ratio (Li and Durbin 2009). In turn, the genome integrity was evaluated using the LAI, which was calculated using LTR_FINDER v.1.0.7 (Xu and Wang 2007), LTR_harvest v.1.5.10 (Ellinghaus et al. 2008), and LTR_retriever v.1.8.0 (Ou and Jiang 2018) with default parameters. Finally, the completeness of the genome assembly was assessed using BUSCO v.5.3.1 (Simão et al. 2015) with default parameters.

### Annotation of repetitive sequences

The repetitive elements were predicted using *ab initio* and homology-based methods. The *ab initio* approach involved the extraction of complete 5′- and 3′-ends of LTR elements using LTR_FINDER v.1.07 (Xu and Wang 2007), LTRharvest v.1.5.10 (Ellinghaus et al. 2008), and LTR_retriever v.1.8.0 (Ou and Jiang 2018) with default parameters. Novel repeat elements were predicted using RepeatModeler v.2.0.10 (Price et al. 2005). The repeat library was downloaded from Repbase v.21.12 (https://www.girinst.org/ downloads/) (Bao et al. 2015). Finally, RepeatMasker v.4.0.7 (Tempel 2012), together with a de novo repeat library and the Repbase database, was used to predict repetitive elements. Tandem repeat was annotated using Tandem Repeat Finder v.4.09 (Benson 1999).

### Gene prediction and functional annotation

Protein-coding genes in the *P. conradinae* genome were predicted using a combination of *ab initio*, homology-, and transcriptome-based strategies. For *ab initio* gene prediction, Augustus v.3.0.3 (Stanke et al. 2006), SNAP v.2006–07-28 (Korf 2004), GenScan v1.0 (Aggarwal and Ramaswamy 2002), and GlimmHMM v.3.0.1 (Majoros et al. 2004) were used for* ab initio* gene prediction based on the repeat-masked genome. We used the sequences of *P. avium* (PRJNA550274, PRJNA419491, PRJNA595502, and PRJNA73727), *Pcer* (PRJNA295439 and PRJNA327561), *P. pseudocerasus* (PRJNA260424), and *P. subhirtella* (PRJNA596558) to perform homology-based predictions using Exonerate v.2.2.0 (Slater and Birney 2005). Transcriptome-based gene models were predicted using StringTie v.1.3.4 (Pertea et al. 2015) and PASA (Haas et al. 2003) based on homologous transcriptomes from Illumina sequencing data (PRJNA260424). These data were then integrated using EvidenceModeler v.1.1.1 (Haas et al. 2008).

Gene functions were predicted based on sequence similarity and domain conservation. This involved using the BLAST tool to search against the NR, KEGG, and Swiss-Prot databases, employing the HMMER v.3.0 to search against Pfam, and using the InterProScan (Jones et al. 2014) to annotate GO terms. Non-coding RNAs (ncRNAs) in the *P. conradinae* genome were predicted using tRNAscan-SE v.1.3.1 (tRNA) (Lowe and Eddy 1997), RNAmmer v.1.2 (rRNA) (Lagesen et al. 2007), and INFERNAL v.1.1.2 (miRNA and snRNA) (Nawrocki et al. 2009). Other ncRNAs were predicted using Rfam v.1.0.4 (Griffiths-Jones et al. 2005).

### Synteny analysis

To explore genome collinearity across species, the Pcon genome was compared with the genomes of Pavi, Pser, and Pper using MUMmer v.3.23 (http://mummer.sourceforge.net). The results of genome collinearity analysis were visualized using MUMmer v.3.23 (http://mummer.sourceforge.net) with default parameters. Furthermore, gene synteny between the eight chromosomes of Pcon, Pavi, Pper, and Pser was determined using Diamond v.2.0.7 (https://github.com/bbuchfink/diamond). Syntenic blocks were generated by comparing the Pcon genome with the Pavi, Pper, and Pser genomes using MCScanX (Wang et al. 2012; https://github.com/wyp1125/MCScanx) with default parameters. The collinearity results were displayed using JCVI (https://github.com/tanghaibao/ jcvi). Finally, structural variations between the genome of Pcon and that of each of the three *Prunus* species were detected using MUMmer v.3.23 and SyRI (Goel et al. 2019).

### Phylogenetic construction, divergence time estimation, and expanded and contracted gene family analysis

To identify orthologous genes, the complete genome sequences of Atha (Zapata et al. 2016), Parm (Groppi et al. 2021), Pavi (Wang et al. 2020b), Pcam (Nie et al. 2023), Pcer (Goeckeritz et al. 2023), *P. dulcis* (Pdul; Alioto et al. 2020), Pfru (Wöhner et al. 2021), *P. huminis* (Phum; https://ngdc.cncb.ac.cn/search/?dbId=gwh&q=GWHBCKI00000000), Pmum (Zheng et al. 2022), Pper (Tan et al. 2021), Ppus (Jiu et al. 2023), Pser (Yi et al. 2020), Pyed (Baek et al. 2018), *Rosa chinensis* (Rchi; Raymond et al. 2018), and Vvin (Jaillon et al. 2007) were retrieved for comparison with Pcon. Gene families were identified using OrthoFinder v.2.2.7 with default parameters. Single-copy orthologous genes were aligned using MUSCLE v.5.1 (Edgar 2004) with default parameters. A ML phylogenetic tree was constructed using PhyML v3.0 with default parameters. The divergence time was estimated using the MCMCtree program in the PAML v.4.9j package (Yano et al. 2016) and the known divergence time from TIMETREE (http://www.timetree.org) was used for calibration. Contracted and expanded gene families were identified using CAFÉ v.3.1 (De Bie et al. 2006).

### Positive selection analysis

In general, the nonsynonymous substitution (*Ka*) to synonymous substitution (*Ks*) rate ratio (ω = *Ka*/*Ks*) is considered a reliable method for assessing the evolution pressures of protein-coding genes (Qu et al. 2019). Single-copy genes from *P. conradinae* and three representative *Prunus* species were aligned using MUSCLE v.5.1, and the alignment results were filtered using Gblocks v.0.91b. The CodeML program in the PAML v.4.9 h package was utilized to infer the most likely *Ka*/*Ks* ratio for each pair of sequences (Nevado et al. 2016). The *Ka*/*Ks* ratio indicates positive selection (ω > 1) (Yang 2007), neutral evolution (ω = 1), or negative purifying selection (0 < ω < 1). The Bayes empirical Bayes (BEB) method was employed to calculate posterior probabilities and identify positively selected sites, after identifying positive selection genes (Yang et al. 2005). Positive selection genes underwent GO and KEGG enrichment analyses using topGO.

### Whole-genome duplication (WGD) and divergence event analysis

The *Ks* values were used to explore the WGD and divergence events between *P. conradinae* and nine other plant species. The timeframe of grapevine fossil records was used as a reference to calculate the WGD event times of other plant species. First, the protein sequences of self or different species were all-*vs.*-all blasted using Diamond v.2.0.7 (https://github.com/bbuchfink/diamond). The best hits of homologous gene pairs were then subjected to MCScanX (Wang et al. 2012) and the respective collinear blocks were identified. Second, the protein sequences of collinear gene pairs were aligned using MUSCLE v.5.1 and converted into codon alignments using ParaAT v.2.0. Finally, *Ks* values were calculated using KaKs Calculator v2.0 (Sun et al. 2022). The Ks density distribution of collinear gene pairs was visualized using ggplot2 in the R package (https://github.com/tidyverse/ggplot2). Collinear blocks from duplication events were classified using the median *Ks* values between homologous genes.

### Phylogenetic and gene cluster analysis of the MADS-box family

Sequence files for the MADS-box gene family (PF00319) were retrieved from the Pfam database (http://pfam.janelia.org/) and TAIR database (https://arabidopsis.org/ index.jsp). MADS-box family members of Atha, Pser, Pyed, and Pper were retrieved from a previous report (Jiu et al. 2023). First, we used the domain file as the initial template to screen all genes using HMMER v.3.3.2 (Johnson et al. 2010) with default parameters. Genes with E-values less than 1e − 5 were retained in *P. conradinae* and *P. avium*. The remaining genes were used as templates for a second screening. Putative genes were identified using BLAST v.2.5.0 to align these sequences with those of the *Arabidopsis* reference genes. MUSCLE v.5.1 was used to generate a high-fidelity sequence alignment of identified genes (Edgar 2004). Furthermore, FastTree v.2.1.11 was used to construct ML phylogenetic trees of the MADS-box gene family (Price et al. 2010). Advanced computational scripts specifically written in Perl were used to map the chromosomal locations of the identified MADS-box genes.

### Supplementary Information

Supplementary Material 1: Figure S1. The estimation of genome size of *Prunus conradinae*. PI positive gated population in a histogram showing PI stained *P. conradinae* (Pcon) and *Solanum lycopersicum* (tomato).

Supplementary Material 2: Figure S2. High-resolution Hi-C contact matrix in the chromosome-level assembly of the *Prunus conradinae* genome. Individual Chrs were scaffolded and independently assembled.

Supplementary Material 3: Figure S3. GO and KEGG pathway enrichment analysis for the expanded gene families in *Prunus conradinae*.

Supplementary Material 4: Figure S4. GO and KEGG pathway enrichment analysis for the contracted gene families in *Prunus conradinae*.

Supplementary Material 5: Figure S5. GO and KEGG pathway enrichment analysis for the unique gene families in *Prunus conradinae*.

Supplementary Material 6: Table S1. Phenotypic characteristics of flowers and fruits in *Prunus conradinae*.

Supplementary Material 7: Table S2. Data statistics of whole-genome sequencing for *Prunus conradinae*. Table S3. The estimation of genome size of *Prunus conradinae* using flow cytometry. Table S4. Statistics for the *Prunus conradinae* assembly. Table S5. Completeness of the genome assembly measured by Benchmarking Universal Single-Copy Orthologs (BUSCO). Table S6. Data statistics of ordering and orienting the scaffolds on 8 pseudomolecules. Table S7. Statistics of repetitive sequence classification from the *Prunus conradinae* genome. Table S8. Completeness of the assembly and the annotated genes measured by Benchmarking Universal Single-Copy Orthologs (BUSCO). Table S9. Statistics of gene functional annotation in the *Prunus conradinae* genome. Table S10. Long terminal repeat assembly index (LAI) analysis and contig N50s of different genome assemblies in *Prunus* species.

Supplementary Material 8: Table S11. Genome syntenic blocks between *Prunus conradinae* and *P. avium*. Table S12. Genome syntenic blocks between *Prunus conradinae* and *P. serrulata*. Table S13. Genome syntenic blocks between *Prunus conradinae* and *P. persica*.

Supplementary Material 9: Table S14. The statistics of Pcon vs. Pavi, and Pcon vs. Pser synteny maps.

Supplementary Material 10: Table S15. Syntenic blocks between *Prunus persica* and *P. avium*. Table S16. Syntenic blocks between *Prunus avium* and *P. serrulata*. Table S17. Syntenic blocks between *Prunus serrulata* and *P. conradinae*.

Supplementary Material 11: Table S18. Structural-variant detection between *Prunus conradinae* and *P*. *avium* genomes. Table S19. Structural-variant detection between *Prunus conradinae* and *P. persica* genomes. Table S20. Structural-variant detection between *Prunus conradinae* and *P. serrulata* genomes.

Supplementary Material 12: Table S21. Comparison of gene faimlies statistics between *Prunus conradinae* and other species.

Supplementary Material 13: Table S22. Gene ontology (GO) enrichment analysis of the expanded gene families in *Prunus conradinae*.

Supplementary Material 14: Table S23. Gene ontology (GO) enrichment analysis of the contracted gene families in *Prunus conradinae*.

Supplementary Material 15: Table S24. Gene ontology (GO) enrichment analysis of the unique gene families in *Prunus conradinae*.

Supplementary Material 16: Table S25. Positively selected orthologous gene pairs between *Prunus avium* and *P. conradinae.*

Supplementary Material 17: Table S26. Positively selected orthologous gene pairs between *Prunus conradinae* and *P. persica*.

Supplementary Material 18: Table S27. Positively selected orthologous gene pairs between *Prunus serrulata* and *P. conradinae*.

Supplementary Material 19: Table S28. The annotation information for transcription factor from positively selected gene pairs (Ka/Ks>1) between *Prunus conadinae* and *P. avium*. Table S29. The annotation information for transcription factor from positively selected gene pairs (Ka/Ks>1) between *Prunus serrulata* and *P. conadinae*. Table S30. The annotation information for transcription factor from positively selected gene pairs (Ka/Ks>1) between *Prunus conadinae* and *P. persica*.

Supplementary Material 20: Table S31. The Ks_peak values and WGD event time of paralogs from Pcon and nine plant species.

Supplementary Material 21: Table S32. The Ks_peak values of orthologs between Pcon and other *Prunus* species.

Supplementary Material 22: Table S33. The members of MADS-box gene family in *Prunus conradinae* and *P. avium.*

Supplementary Material 23: Table S34. MADS-box genes microsyntenic blocks between *Prunus conradinae* and *P. serrulata*.

## Data Availability

All data supporting the results of this study are included in the manuscript and its additional files. The raw data of *Prunus conradinae* genome are available at figshare (10.6084/m9.figshare.25435240.v2).

## Abbreviations

AG: AGAMOUS
AGL24: AGAMOUS-like 24
ANR1: ARABIDOPSIS NITRATE REGULATED 1
AP1: APETALA1
AP3: APETALA3
Atha: *Arabidopsis thaliana*
BUSCO: Benchmarking Universal Single-Copy Ortholog
COG: Clusters of Orthologous Groups of proteins
FLC: FLOWERING LOCUS C
GO: Gene ontology
Hi-C: High-throughput chromosome conformation capture
KEGG: Kyoto Encyclopedia of Genes and Genomes
LAI: Long terminal repeat assembly index
LTR-RTs: Long terminal repeat retrotransposons
ML: Maximum-likelihood
Mya: Million years ago
NGS: Next-generation sequencing
NR: Non-redundant
ONT: Oxford Nanopore Technology
Parm: *Prunus armeniaca*
Pavi: *Prunus avium*
Pcam: *Prunus campanulata*
Pcer: *Prunus cerasus*
Pcon: *Prunus conradinae*
Pdul: *Prunus dulcis*
Pfru: *Prunus fruticosa*
Phum: *Prunus huminis*
PI: PISTILLATA
Pmum: *Prunus mume*
Pper: *Prunus persica*
Ppus: *Prunus pusilliflora*
Pser: *Prunus serrulata*
Pyed: *Prunus yedoensis*
Rchi: *Rosa chinensis*
SEP: SEPALLATA
SOC1: SUPPRESSOR OF OVEREXPRESSION OF CONSTANS 1
STK: SEEDSTICK
*SVP*: *SHORT VEGETATIVE PHASE*
SyRI: Synteny and Rearrangement Identifier
TAIR: The Arabidopsis Information Resource
TEs: Transposable elements
TFs: Transcription factors
TM8: TOMATO MADS-box 8
TT16: TRANSPARENT TESTA 16
Vvin: *Vitis vinifera*
WGD: Whole genome duplication
