# Supplementary figures and images for: Chromosome-level genome assembly provides insights into the genetic diversity, evolution, and flower development of Prunus conradinae
Source: Mol Hortic. 2024 Jun 19;4:25. doi: 10.1186/s43897-024-00101-7 (PMC11186256; doi:10.1186/s43897-024-00101-7)

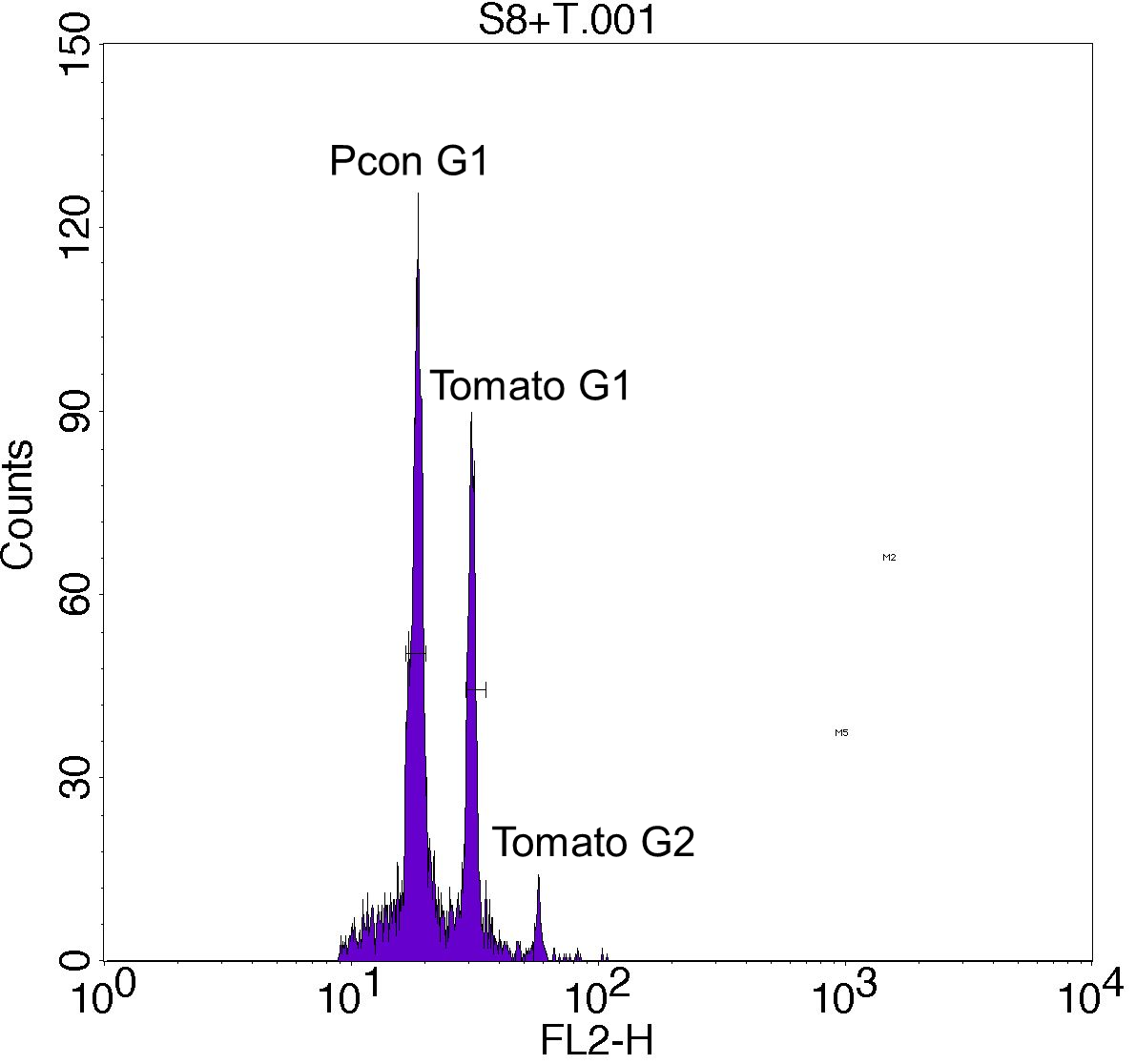

Supplement: Supplementary file 1 — Supplementary Material 1: Figure S1. The estimation of genome size of Prunus conradinae. PI positive gated population in a histogram showing PI stained P. conradinae (Pcon) and Solanum lycopersicum (tomato). [file 43897_2024_101_MOESM1_ESM.tif]

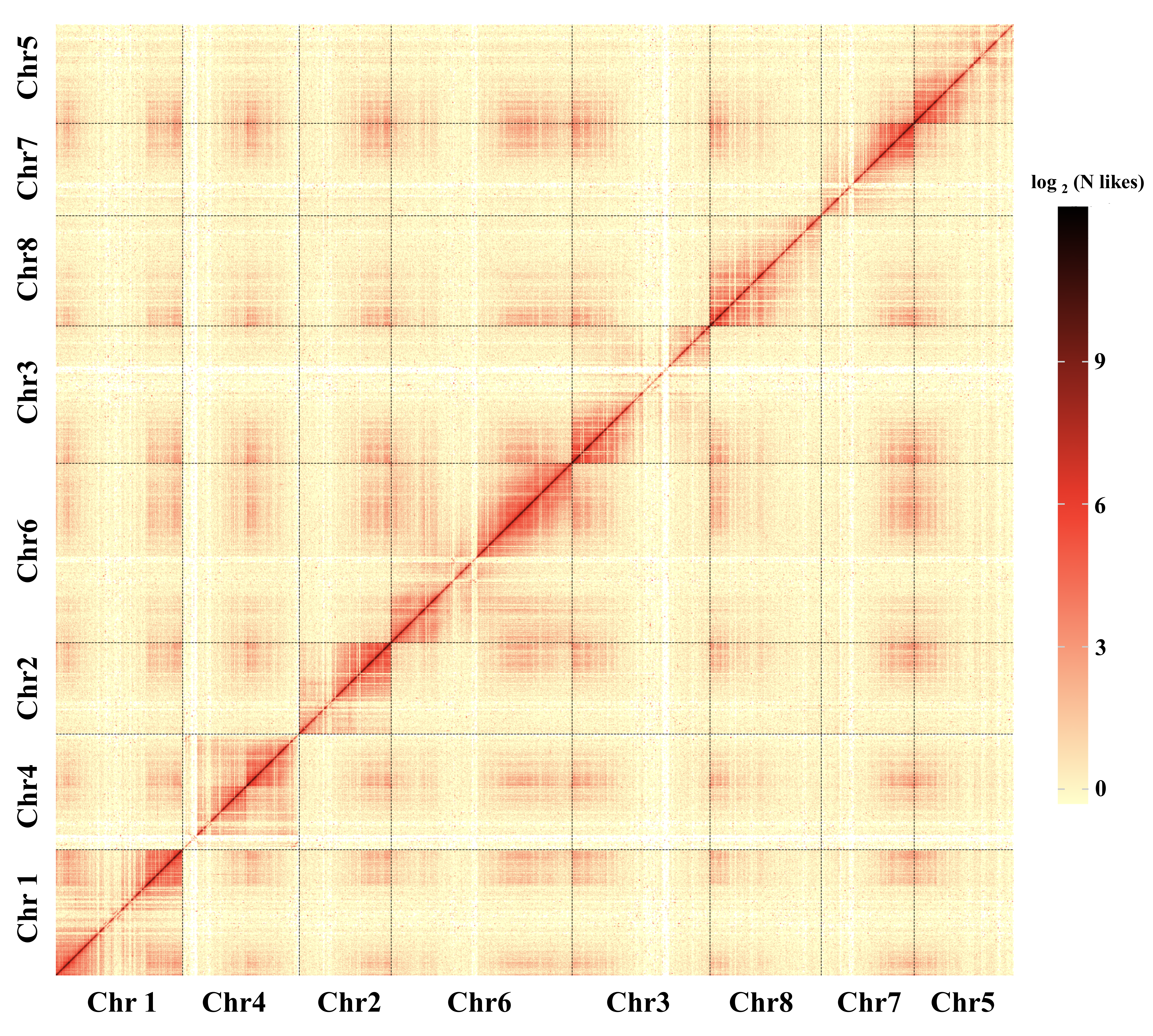

Supplement: Supplementary file 2 — Supplementary Material 2: Figure S2. High-resolution Hi-C contact matrix in the chromosome-level assembly of the Prunus conradinae genome. Individual Chrs were scaffolded and independently assembled. [file 43897_2024_101_MOESM2_ESM.tif]

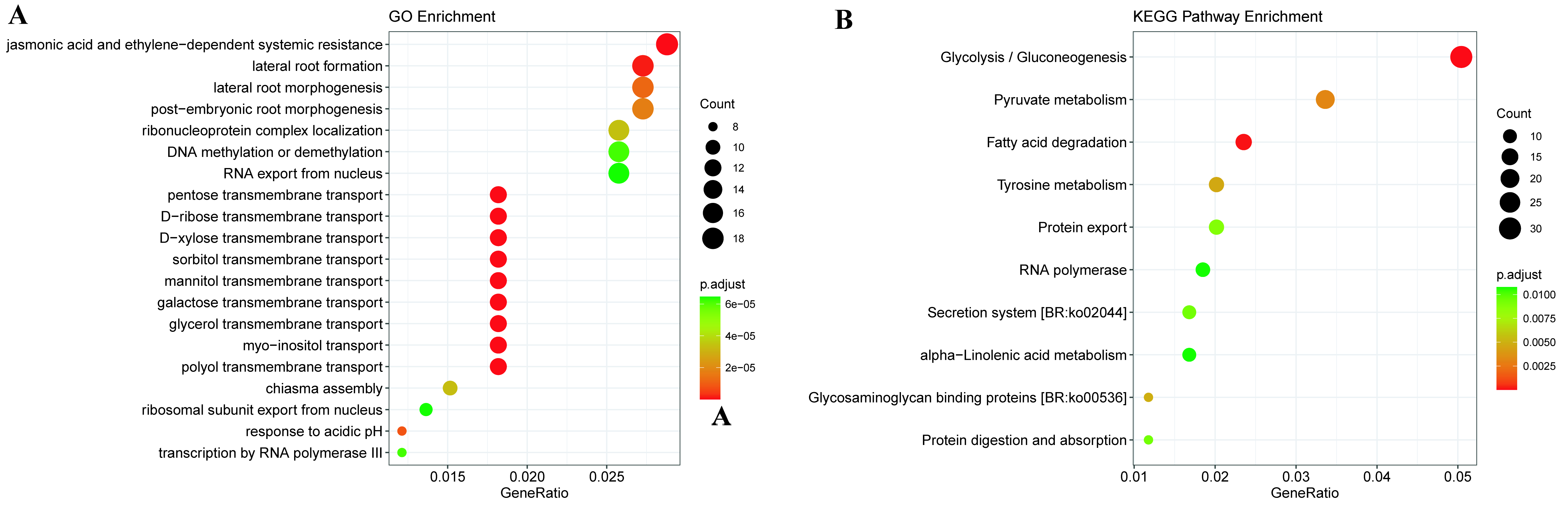

Supplement: Supplementary file 3 — Supplementary Material 3: Figure S3. GO and KEGG pathway enrichment analysis for the expanded gene families in Prunus conradinae. [file 43897_2024_101_MOESM3_ESM.tif]

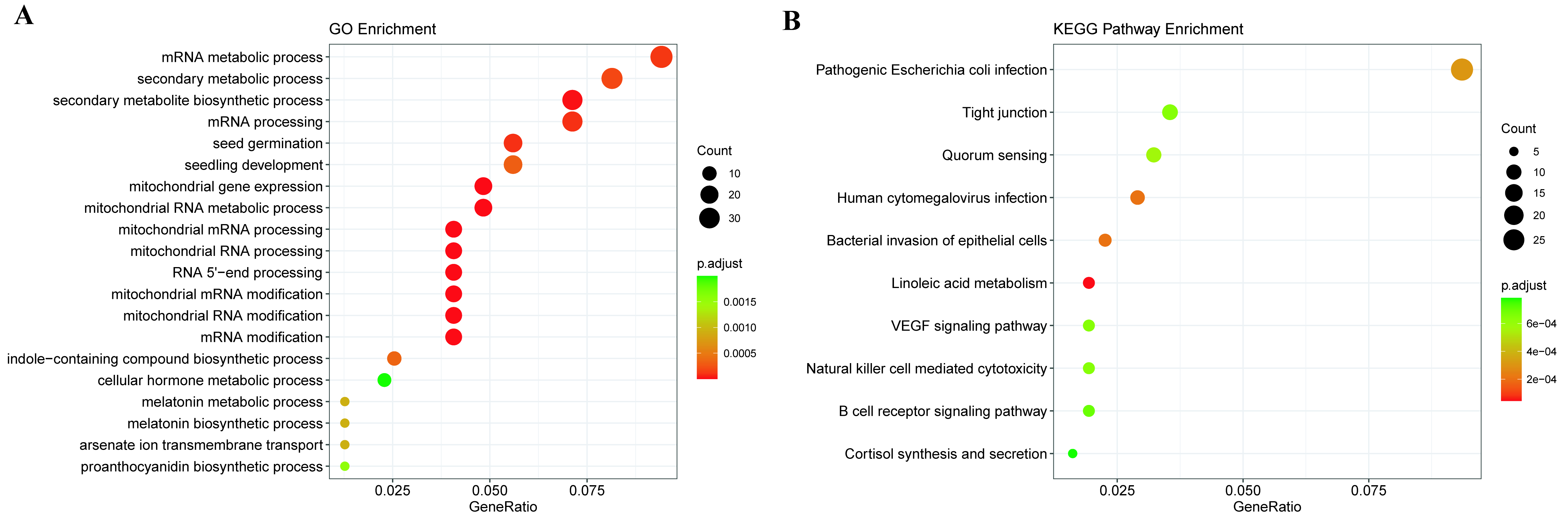

Supplement: Supplementary file 4 — Supplementary Material 4: Figure S4. GO and KEGG pathway enrichment analysis for the contracted gene families in Prunus conradinae. [file 43897_2024_101_MOESM4_ESM.tif]

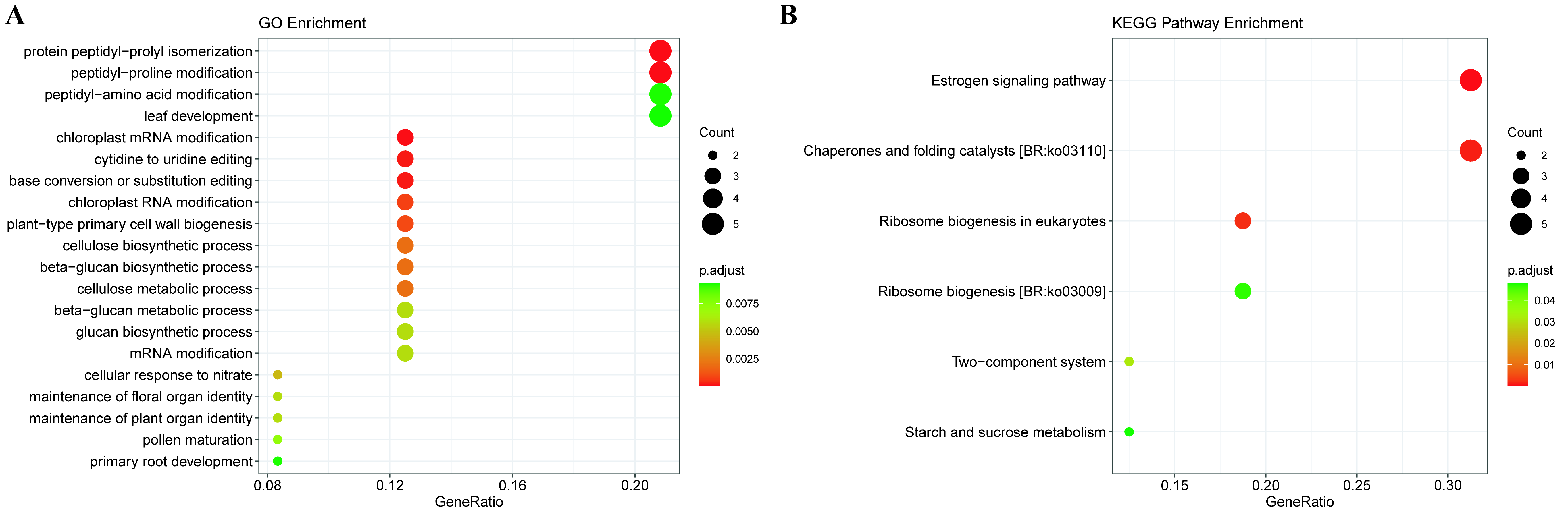

Supplement: Supplementary file 5 — Supplementary Material 5: Figure S5. GO and KEGG pathway enrichment analysis for the unique gene families in Prunus conradinae. [file 43897_2024_101_MOESM5_ESM.tif]
