# Supplementary material for: Chromosome-level genome assembly provides insights into the genetic diversity, evolution, and flower development of Prunus conradinae
Source: Mol Hortic. 2024 Jun 19;4:25. doi: 10.1186/s43897-024-00101-7 (PMC11186256; doi:10.1186/s43897-024-00101-7)
Supplement: Supplementary file 6 — Supplementary Material 6: Table S1. Phenotypic characteristics of flowers and fruits in Prunus conradinae. [file 43897_2024_101_MOESM6_ESM.docx]

**Table S1. Phenotypic characteristics of flowers and fruits in *Prunus conradinae*.**

| No. | Stamen number | Pedicel length  (cm) | Flower numbers of each inflorescence | Petals numbers of each flower | Fruit stalk length (mm) | Fruit stalk diameter (mm) | Titratable acid (TA) (%) | Total soluble solids (%) | Fruit vertical diameter (mm) | Fruit transverse diameter (broadside) (mm) | Fruit transverse diameter (narrowside) (mm) | Fruit weight (g) | Hardness (g) |
| --- | --- | --- | --- | --- | --- | --- | --- | --- | --- | --- | --- | --- | --- |
| 1 | 33 | 2.2 | 5 | 5 | 12.75 | 0.87 | 0.938 | 20.6 | 9.68 | 9.65 | 7.57 | 0.60 | 54.878 |
| 2 | 34 | 1.9 | 4 | 5 | 17.24 | 1.05 | 0.804 | 21.7 | 9.75 | 9.04 | 7.84 | 0.52 | 77.103 |
| 3 | 28 | 2.2 | 4 | 5 | 13.20 | 1.03 | 0.804 | 21.6 | 9.44 | 9.93 | 8.19 | 0.56 | 64.362 |
| 4 | 33 | 1.8 | 3 | 5 | 13.78 | 1.02 | 0.871 | 24.7 | 10.03 | 8.92 | 8.29 | 0.60 | 64.546 |
| 5 | 25 | 2.3 | 5 | 5 | 13.06 | 0.96 |  | 22.7 | 10.13 | 9.67 | 8.49 | 0.63 | 54.728 |
| 6 | 31 | 1.9 | 2 | 5 | 15.13 | 1.04 |  | 21.7 | 10.36 | 10.24 | 9.22 | 0.65 | 57.536 |
| 7 | 38 | 1.9 | 4 | 5 | 15.97 | 0.96 |  | 28.7 | 9.74 | 9.75 | 8.65 | 0.63 | 61.841 |
| 8 | 40 | 2.0 | 5 | 5 | 13.65 | 0.99 |  | 21.9 | 9.78 | 9.44 | 7.59 | 0.51 | 60.782 |
| 9 | 33 | 1.8 | 4 | 5 | 15.34 | 0.92 |  | 24.3 | 8.97 | 9.45 | 8.41 | 0.65 | 61.818 |
| 10 | 37 | 2.3 | 4 | 5 | 13.29 | 0.90 |  | 23.5 | 9.88 | 9.98 | 8.55 | 0.62 | 62.428 |
| 11 | 38 | 1.9 | - | 5 | 13.98 | 0.95 |  | 24.8 | 10.51 | 9.56 | 8.52 | 0.57 | 71.417 |
| 12 | - | 1.8 | - | 5 | 13.30 | 0.83 |  | 21.2 | 9.10 | 9.03 | 7.84 | 0.47 | 67.792 |
| 13 | - | 2.0 | - | 5 | 12.90 | 0.95 |  | 23.8 | 9.99 | 9.54 | 8.57 | 0.58 | 91.732 |
| 14 | - | 1.9 | - | 5 | 12.81 | 0.81 |  | 21.9 | 10.28 | 9.77 | 9.08 | 0.67 | 76.182 |
| 15 | - | 2.2 | - | 5 | 13.23 | 0.87 |  | 25.3 | 9.89 | 9.65 | 7.90 | 0.51 | 101.434 |
| 16 |  |  |  |  | 13.83 | 0.95 |  | 22.6 | 9.65 | 8.97 | 8.26 | 0.52 |  |
| 17 |  |  |  |  | 15.24 | 0.86 |  | 27.8 | 9.61 | 9.25 | 8.40 | 0.57 |  |
| 18 |  |  |  |  | 14.26 | 0.85 |  | 20.9 | 9.53 | 9.40 | 9.13 | 0.61 |  |
| 19 |  |  |  |  | 14.97 | 0.92 |  | 22.8 | 8.35 | 9.09 | 7.68 | 0.44 |  |
| 20 |  |  |  |  | 13.25 | 1.06 |  | 24.1 | 10.57 | 10.15 | 9.00 | 0.66 |  |
| 21 |  |  |  |  | 12.72 | 0.87 |  |  | 9.69 | 9.12 | 8.25 | 0.51 |  |
| 22 |  |  |  |  | 15.93 | 0.94 |  |  | 9.78 | 9.18 | 8.62 | 0.57 |  |
| 23 |  |  |  |  | 13.99 | 0.75 |  |  | 9.72 | 9.38 | 8.56 | 0.53 |  |
| 24 |  |  |  |  | 15.57 | 0.90 |  |  | 9.92 | 9.35 | 8.59 | 0.51 |  |
| 25 |  |  |  |  | 12.98 | 0.99 |  |  | 10.07 | 9.98 | 8.94 | 0.65 |  |
| 26 |  |  |  |  | 12.41 | 1.16 |  |  | 9.92 | 8.88 | 8.21 | 0.46 |  |
| 27 |  |  |  |  | 13.50 | 0.97 |  |  | 9.98 | 9.57 | 8.45 | 0.62 |  |
| 28 |  |  |  |  | 13.37 | 1.09 |  |  | 9.91 | 9.70 | 8.49 | 0.61 |  |
| 29 |  |  |  |  | 14.29 | 0.98 |  |  | 9.69 | 9.87 | 9.11 | 0.62 |  |
| 30 |  |  |  |  | 14.14 | 0.75 |  |  | 9.66 | 9.79 | 8.78 | 0.56 |  |
| 31 |  |  |  |  | 12.40 | 0.78 |  |  | 9.88 | 9.94 | 8.77 | 0.55 |  |
| 32 |  |  |  |  | 13.77 | 0.95 |  |  | 10.10 | 9.03 | 8.29 | 0.55 |  |
