# Supplementary material for: Chromosome-level genome assembly provides insights into the genetic diversity, evolution, and flower development of Prunus conradinae
Source: Mol Hortic. 2024 Jun 19;4:25. doi: 10.1186/s43897-024-00101-7 (PMC11186256; doi:10.1186/s43897-024-00101-7)
Supplement: Supplementary file 7 — Supplementary Material 7: Table S2. Data statistics of whole-genome sequencing for Prunus conradinae. Table S3. The estimation of genome size of Prunus conradinae using flow cytometry. Table S4. Statistics for the Prunus conradinae assembly. Table S5. Completeness of the genome assembly measured by Benchmarking Universal Single-Copy Orthologs (BUSCO). Table S6. Data statistics of ordering and orienting the scaffolds on 8 pseudomolecules. Table S7. Statistics of repetitive sequence classification from the Prunus conradinae genome. Table S8. Completeness of the assembly and the annotated genes measured by Benchmarking Universal Single-Copy Orthologs (BUSCO). Table S9. Statistics of gene functional annotation in the Prunus conradinae genome. Table S10. Long terminal repeat assembly index (LAI) analysis and contig N50s of different genome assemblies in Prunus species. [file 43897_2024_101_MOESM7_ESM.docx]

**Table S2. Data statistics of whole-genome sequencing for *Prunus conradinae*.**

| libraries | Total data (bp) | Read length (bp) |
| --- | --- | --- |
| Illumina | 74,362,904,100 | 150 |
| Nanopore | 44,969,401,913 | - |
| Hi-C | 90,910,744,800 | 150 |
| Total | 210,243,050,813 | - |

**Table S3. The estimation of genome size of *Prunus conradinae* using flow cytometry.**

| Sample code | Internal reference species | Fluorescence intensity of tomato | Fluorescence intensity of C6 | | Ratio | Haplotype genome size (Mb) | Tetraploid genome size (Mb) |
| --- | --- | --- | --- | --- | --- | --- | --- |
| C6 | Solanum lycopersicum | 30.44 | 18.05 | 0.59 | | 266.835 | 1067.34 |

**Table S4. Statistics for the *Prunus conradinae* assembly.**

| Sample ID | Length (bp) | | Number | |
| --- | --- | --- | --- | --- |
|  | Contig | Scaffold | Contig | Scaffold |
| Total | 289,583,391 | 289,626,891 | 113 | 26 |
| Max | 14,803,222 | 52,777,529 | - | - |
| N50 | 4,471,996 | 34,173,511 | 21 | 4 |

Note: N50 refers to the size above which 50% of the total length of the sequence assembly can be found.

**Table S5. Completeness of the genome assembly measured by Benchmarking Universal Single-Copy Orthologs (BUSCO).**

| Description | Assembly | |
| --- | --- | --- |
|  | Number | Percentage (%) |
| Complete BUSCOs | 1566 | 97.1 |
| Complete and single-copy BUSCOs | 1531 | 94.9 |
| Complete and duplicated BUSCOs | 35 | 2.2 |
| Fragmented BUSCOs | 10 | 0.6 |
| Missing BUSCOs | 38 | 2.3 |
| Total BUSCO groups searched | 1614 | - |

**Table S6. Data statistics of ordering and orienting the scaffolds on 8 pseudomolecules.**

| Chromosome | Length (bp) | N_base | Gap_ratio (%) |
| --- | --- | --- | --- |
| Chr1 | 52,777,529 | 6500 | 0.01 |
| Chr2 | 40,421,504 | 4500 | 0.01 |
| Chr3 | 34,173,511 | 4000 | 0.01 |
| Chr4 | 32,482,755 | 5500 | 0.02 |
| Chr5 | 26,797,334 | 2500 | 0.01 |
| Chr6 | 37,020,845 | 6500 | 0.02 |
| Chr7 | 27,120,995 | 4000 | 0.01 |
| Chr8 | 29,071,908 | 6500 | 0.02 |
| Total | 279,866,381 | 43500 | 0.02 |
|  |  |  |  |

**Table S7. Statistics of repetitive sequence classification from the *Prunus conradinae* genome.**

| Type | Length (bp) | Rate (%) |
| --- | --- | --- |
| **Class I: Retrotransposon** | **76,664,574** | **27.39%** |
| **LTR-Retrotransposon** | **72,435,797** | **25.88%** |
| LTR/*Copia* | 28,127,716 | 10.05% |
| LTR/*Gypsy* | 41,927,429 | 14.98% |
| LTR-other | 2,380,652 | 0.85% |
| **Non-LTR Retrotransposon** | **4,228,777** | **1.51%** |
| SINE | 36,211 | 0.01% |
| LINE | 4,192,566 | 1.50% |
| **Class II: DNA Transposon** | **28,804,474** | **10.29%** |
| EnSpm/CACTA | 11,265,183 | 4.03% |
| Harbinger | 3,625,179 | 1.30% |
| Helitron | 3,210,708 | 1.15% |
| MuDR | 2,957,653 | 1.06% |
| Tcl/Mariner | 142,635 | 0.05% |
| hAT | 6,186,700 | 2.21% |
| DNA-other | 1,416,416 | 0.51% |
| **Low Complexity** | **26,884** | **0.01%** |
| **Tandem repeat** | **8,613,370** | **3.08%** |
| **Unclassified** | **15,273,487** | **5.46%** |
| **Total content** | **129,382,789** | **46.23%** |

**Table S8. Completeness of the assembly and the annotated genes measured by Benchmarking Universal Single-Copy Orthologs (BUSCO).**

| Description | Genome | | Proteins | |
| --- | --- | --- | --- | --- |
|  | Number | Percentage (%) | Number | Percentage (%) |
| Complete BUSCOs | 1566 | 97.1 | 1520 | 94.2 |
| Complete and single-copy BUSCOs | 1531 | 94.9 | 696 | 43.1 |
| Complete and duplicated BUSCOs | 35 | 2.2 | 824 | 51.1 |
| Fragmented BUSCOs | 10 | 0.6 | 41 | 2.5 |
| Missing BUSCOs | 38 | 2.3 | 53 | 3.3 |
| Total BUSCO groups searched | 1614 | - | 1614 | - |

**Table S9. Statistics of gene functional annotation in the *Prunus conradinae* genome.**

| Database | Annotated Number | Annotated Percent (%) |
| --- | --- | --- |
| NR | 30,570 | 98.19% |
| Swiss-Prot | 21,205 | 68.11% |
| TAIR | 24,116 | 77.46% |
| MSU | 24,692 | 79.31% |
| GO | 9,742 | 31.29% |
| KEGG | 12,084 | 38.81% |
| COG | 26,018 | 83.57% |
| eggNOG | 26,018 | 83.57% |
| Pfam | 21,017 | 67.51% |
| Annotated | 30,580 | 98.22% |
| Total | 31,134 | 100.00% |

**Table S10**. **Long terminal repeat assembly index (LAI) analysis and contig N50s of different genome assemblies in *Prunus* species.**

| Species | LAI | Contig N50 (Mb) |
| --- | --- | --- |
| *P. conradinae* | 18.26 | 4.47 |
| *P. avium*^1^ | 19.68 | 3.25 |
| *P. persica*^2^ | 18.79 | 0.25 |
| *P. armeniaca*^3^ | 16.29 | 1.02 |
| *Prunus pusilliflora*^4^ | 17.35 | 6.00 |
| *P. yedoensis*^5^ | 6.87 | 0.92 |
| *P. domestica*^6^ | 2.27 | 1.74 |

**References:**

1. **Wang, J., Liu, W., Zhu, D., Hong, P., Zhang, S., Xiao, S., Tan, Y., Chen, X., Xu, L., Zong, X.,** **et al.** (2020b). Chromosome-scale genome assembly of sweet cherry (*Prunus avium* L.) cv. Tieton obtained using long-read and Hi-C sequencing. Hortic. Res. **7(1)**:122.
2. **Verde, I., Jenkins, J., Dondini, L., Micali, S., Pagliarani, G., Vendramin, E., Paris, R., Aramini, V., Gazza, L., Rossini, L., et al.（2017）**The Peach v2.0 release: high-resolution linkage mapping and deep resequencing improve chromosome-scale assembly and contiguity**.** BMC Genomics **18(1)**:225.
3. **Jiang, F., Zhang, J., Wang, S., Yang, L., Luo, Y., Gao, S., Zhang, M., Wu, S., Hu, S., Sun, H., et al.** (2019). The apricot (*Prunus armeniaca* L.) genome elucidates Rosaceae evolution and beta-carotenoid synthesis. Hortic. Res. **6**:128.
4. **Jiu, S., Chen, B., Dong, X., Lv, Z., Wang, Y., Yin, C., Xu, Y., Zhang, S., Zhu, J., Wang, J., et al. (2023).** Chromosome-scale genome assembly of *Prunus pusilliflora* provides novel insights into genome evolution, disease resistance, and dormancy release in *Cerasus* L. Hortic. Res. **10(5)**: uhad062.
5. **Baek, S., Choi, K., Kim, G.B., Yu, H.J., Cho, A., Jang, H., Kim, C., Kim, H.J., Chang, K.S., Kim, J.H., et al.** (2018). Draft genome sequence of wild *Prunus yedoensis* reveals massive inter-specific hybridization between sympatric flowering cherries. Genome Biol. **19:**1–17.
6. **Zhebentyayeva, T., Shankar, V., Scorza, R., Callahan, A., Ravelonandro, M., Castro, S., DeJong, T., Saski, C.A., and Dardick, C.** (2019). Genetic characterization of worldwide *Prunus domestica* (plum) germplasm using sequence-based genotyping. Hortic. Res. **6**:12.
